# Systematic profiling of the effective ingredients and mechanism of *Scabiosa comosa* and *S. tschilliensis* against hepatic fibrosis combined with network pharmacology

**DOI:** 10.1038/s41598-021-81399-x

**Published:** 2021-01-28

**Authors:** Qianwen Chen, Yuanyuan Wang, Feixiang Ma, Mengdi Han, Zhen Wang, Peifeng Xue, Jingkun Lu

**Affiliations:** 1 Department of Basic Medicine, Medical University of Inner Mongolia, Jinshan Development Zone, Hohhot, Inner Mongolia China; 2 Department of Pharmacy, Medical University of Inner Mongolia, Jinshan Development Zone, Hohhot, Inner Mongolia China

**Keywords:** Cell biology, Drug discovery, Molecular biology, Structural biology

## Abstract

*Scabiosa comosa* and *S. tschilliensis* (SCST) are traditionally used for liver diseases in Mongolian medicine. However, their active ingredients and molecular mechanisms are unknown. The present study employed network pharmacology and experimental verification approaches to decipher the common pharmacological mechanisms of SCST on liver fibrosis, which is the key step in liver diseases. We predicted the targets of all available SCST ingredients with the SWISS and SuperPred servers and clustered the targets related to liver fibrosis from DrugBank, the OMIM database and the literature. We further evaluated the links between the herbal ingredients and pharmacological actions to explore the potential mechanism of action of SCST. We found that the PPARG signalling pathway could be regulated by SCST for liver fibrosis through enrichment analysis. The key targets included 8 co-targets, including HSP90AA1, PPARG, HSP90AB1, STAT1, etc., which play pivotal roles in the pathogenesis of liver fibrosis. Additionally, the top 15 key compounds included flavonoids and phenylpropanoids. Central to the pathogenesis of liver fibrosis is trans-differentiation or activation of hepatic stellate cells (HSCs). Therefore, LX2 cells, an immortalized human HSC line, were studied. Here, a total 37 components were isolated and identified from the inflorescences of SCST, including the new compound tschilliensisin, and the first separated components, β-sitosterol and luteolin, and these compounds were assessed against anti-hepatic fibrosis. An MTT assay and quantitative real-time polymerase chain reaction (qRT-PCR) and Western blotting analyses demonstrated that the flavonoids of SCST revealed anti-hepatic fibrosis effects via anti-proliferation and increases in the *Stat1, Pparg, Hsp90aa1* genes and STAT1 and PPARG proteins in LX-2 cells. In conclusion, these results indicate that SCST has multi-targeted and multi-component synergistic anti-hepatic fibrosis effects.

## Introduction

Hepatic fibrosis is a pathological consequence of chronic liver diseases, which can ultimately lead to cirrhosis and hepatocellular carcinoma^1,2^. The key stage of hepatic fibrosis is the activation of hepatic stellate cells (HSCs), which undergo obvious phenotypic alterations, including increased cell proliferation, α-SMA expression and ECM production^3,4^.

In the normal liver, quiescent HSCs (qHSCs) are lipid-containing cells that store retinoids and express higher levels of lipid-related genes, such as peroxisome proliferator-activated receptor gamma (PPARG). Under chronic liver injury, qHSCs are transformed into activated hepatic stellate cells (aHSCs) and gradually lose their fat-storing phenotype^5,6^.

*Scabiosa comosa* and *S. tschilliensis* (SCST), ‘Lanpenhua’ in Chinese, are the dry inflorescences of *Scabiosa comosa Fisch. Ex Roem. et Schult* and *Scabiosa tschilliensis Grunning* that are traditionally used to treat liver diseases in Mongolian medicine. It is clinically used to treat diseases such as pulmonary heat, liver heat, throat heat, headache, fever, cough, and jaundice^7^. It was reviewed in the literature^7^ that SCST showed some pharmacological actions, such as fever relief, anti-inflammatory, antioxidative, renal ischaemia reperfusion injury protection, platelet aggregation inhibition, conscious sedation, and immune function enhancement. SCST was found to be rich in flavonoids, triterpenoids, iridoids, coumarins, organic acids and volatile oils. To date, our team has isolated and identified 22 compounds from SCST, including the new compound tschilliensisin and the first isolated components, β-sitosterol and luteolin^7,8^, and further identified or tentatively characterized a total of 17 absorbed prototype constituents and 22 metabolites found in rat plasma after oral administration. The possible metabolic pathways of these constituents involved sulfation, glucuronidation, demethylation, hydroxylation, and so on^9^.

Recently, TCM network pharmacology was proposed by Li et al.^10–14^, which combine the TCM theory with the interactive network to visualize the relationship between herbal formulae and diseases^15,16^. The combinatorial rules and holistic regulation effects of herbal formulae be conveyed^16–19^. Therefore, TCM network pharmacology can be used to understand the scientific basis of TCM herbal formulae at the molecular level and from a systemic perspective.

In the present study, we employed integrated network pharmacology to profile a network map of bioactive ingredients and molecular targets for the anti-hepatopathy mechanism of SCST. Then, 36 components of SCST, mainly flavonoids, phenylpropanoids and iridoids, were assessed for their activity and verified targets with HSCs closely related to liver fibrosis. To our knowledge, this is the first study to reveal the comprehensive results of the active ingredients and molecular mechanisms of SCST in vitro and to indicate that SCST has multi-targeted and multi-component synergistic anti-hepatic fibrosis effects.

## Materials and methods

### Network pharmacology analysis

A total of 73 compounds from SCST were obtained from the TCMSP (http://lsp.nwu.edu.cn/index.php) and published literature^7–9,20–38^. Information on these compounds is listed in Supplementary Table S1. Excluding the essential oil, compounds that met the properties for oral bioavailability (OB) (≥ 20%), drug-likeness (DL) (≥ 0.18) and Lipinski’s rules or that have definite pharmacological activity were selected as candidate ingredients. The chemical structures of the SCST ingredients were drawn with ChemBioDraw 14.0 and transformed into Mol2 format. Swiss (http://www.swisstargetprediction.ch/) (score ≥ 0.7) and SuperPred (http://prediction.charite.de/) (known ligand-target) were used to predict the potential targets. Hepatic fibrosis-related genes were obtained from OMIM (http://www.omim.org/) and DRUGBANK (https://www.drugbank.ca/), and protein–protein interactions (PPIs) were assessed using STRING (https://string-db.org/cgi/input.pl) (minimum required interaction score ≥ 0.7). DAVID (https://david.ncifcrf.gov/, version 2013) was used for GO and KEGG pathway clustering analyses (*P* < 0.01, Benjamini method). Based on the above results, the compound-target-pathway network was constructed using Cytoscape (version 3.5.1; http://www.cytoscape.org/). The topological parameters, including *degree, betweenness, and closeness,* were analysed.

### Molecular docking

Through the analysis of the above-mentioned Network Analyser plug-in and the prediction results of DAVID, the important signal pathways/key targets and main active components in the treatment of liver fibrosis in the network were analysed, and the SCST components were molecularly docked with the corresponding target proteins. Key proteins and their co-crystals were screened and downloaded from the RCSB Protein Data Bank (http://www.rcsb.org/pdb/home/home.do) website and imported into Leadit to prepare the protein and co-crystals. Water molecules were deleted, polar hydrogen atoms were added, and the loop structure was complete. The Schrodinger molecular docking program was used to study the molecular docking between the compound and the active site of the target structure. Discovery Studio 2016 Client is was to analyse the Schrodinger docking results to investigate the binding mode and the binding free energy of the compound and the corresponding target to determine the affinity of the compound for the target.

### Drugs and Reagents

The 36 compounds found in SCST were collected, of which 22 were isolated and identified in our laboratory, and the other 14 were purchased from Co. SaiBaiCao (Beijing, China). The 36 monomers were dissolved separately in DMSO and tested at concentrations of 12.5–400 μM. In all of the dilutions, the concentration of DMSO did not exceed 0.1%.

### Cell culture and reagents

LX2 cells, an immortalized human HSC line, were provided by Beina Chuanglian Biotechnology Research Institute (Beijing, China). The cells were maintained with 10% or 1% foetal bovine serum in DMEM supplemented with 10% (activated culture) or 2% (quiesced culture) foetal bovine serum (FBS, PAN), 100 IU/ml penicillin, and 100 μg/ml streptomycin (Gibco, 15140-122). Cells were incubated in a 5% CO_2_ humidified atmosphere at 37 °C.

### Lipid staining

A fat-storing phenotype is a distinct feature of qHSCs^39^. LX-2 cells were seeded in 6-well plates at a density of 2 × 10^5^/well and incubated for 24 h before the addition of the stimulus. For identification of the activated phenotypes, LX-2 cells were cultured with 10% FBS (for aHSCs) or 2% FBS (for qHSCs) for 48 h, subsequently treated or not for 24 h with 20 µM of the PPARγ agonist rosiglitazone (RSG) (Sigma, R2408), and then fixed with 16% formalin in PBS for 0.5 h. Oil Red O (0.5% w/v in isopropanol) (Solarbio, G1262) was diluted with a 67% volume of water, filtered, and added to the fixed HSCs. Images were randomly captured using an objective lens (10 ×) in 3 different sections. After removing the staining solution, the dye retained in the cells was eluted into isopropanol, and the optical density (OD) was measured with a microplate reader (ThermoFisher Multiskan FC, USA) at 510 nm.

### Cell proliferation assay

LX-2 cells were seeded in 96-well plates at a density of 1 × 10^4^/well. After incubation at 37 °C for 24 h, the cells were treated with 36 monomers of SCST in the dose range of 12.5–400 μM for another 24 h. Detailed information on these 37 SCST monomers is listed in Supplementary Table S2. Cell viability was evaluated by 3-(4,5-dimethylthiazol-2-yl)-2,5 diphenyltetrazolium bromide (MTT) assay as previously described. Treated cells were incubated with a 10% MTT solution at 37 °C for 4 h, the supernatants were removed, and the formazan crystals were dissolved in 150 µl of DMSO per well. The absorbance was measured on a microplate reader at 490 nm. The relative percentage of cell viability was expressed as a percentage of that of the control cells.

### Real time quantitative PCR

Total RNA was extracted from cells using TRIzol reagent (TianGen, DP424) according to the manufacturer’s protocol. Total RNA was reverse transcribed into cDNA with a reverse transcription assay kit (TianGen, China). *α-Sma, Stat1, Hsp90aa1* and *Pparg* primer sequences were designed with NCBI Gene Bank (https://www.ncbi.nlm.nih.gov) and Primer 5.0 software. Oligo synthesis was performed by Sangon Biotech Co. Ltd. (Shanghai, China). *Gapdh* was used to normalize gene expression. Sequences of the specific primer sets are as follows: *Gapdh* (NM-014364), forward, 5′- CAATGCCTCCTGCACCACCAACTGC -3′, reverse, 5′-GCAGTTGGTGGTGCAGGAGGCATTG-3′; *Stat1,* forward, 5′-GGTAATTGACCTCGAGACGACCTCTC-3′, reverse, 5′-GGAAGAAGGACAGATTCCTGGGTTCC-3′; *Pparg,* forward, 5′-TCTCTCCGTAATGGAAGACC-3′, reverse, 5′-GCATTATGAGACATCCCCAC-3′; *Hsp90aa1,* forward, 5′-AAGTTGAAAAGGTGGTTGTG-3′, reverse, 5′-AATAATGGAATGGTCAGGGT-3’.

### Western blotting

Cell samples were homogenized with cell lysis buffer for Western blotting and IP (1% PMSF) (Beyotime, P0013; Biosynthesis Inc, C05-01002) for 30 min. Total protein from the samples was separated by 10% SDS–polyacrylamide gel electrophoresis (SDS-PAGE) and then transferred to nitrocellulose blotting (NC) membranes. After blocking with 5% non-fat milk for 1 h at room temperature, the membranes were incubated with rabbit monoclonal anti-STAT1 (1:1000, CST:#14994, USA), PPARG (1:1000, CST:#2443, USA), P-STAT1 (1:1000, CST:#9167, USA) and rabbit anti-GAPDH (1:1000, CST: #2118, USA) primary antibodies overnight at 4 °C and then incubated with secondary antibodies (1:2000, BOSTER: BA1054, China) at room temperature for 1 h. The bands were detected by enhanced chemiluminescent ECL reagents (Absin Bio: abs920, China). GAPDH was used as an internal control.

### Statistical analysis

GraphPad Prism 5.0 (San Diego, CA, USA) was used for statistical analyses. Data are presented as the means ± SD. Two-way ANOVA and one-way ANOVA were used to compare significant differences. For statistical comparison of the means between two groups, unpaired two-tailed Student’s *t*-test was carried out. A *P* value of less than 0.05 was considered statistically significant.

## Results

### Network pharmacology analysis

**Figure 1:**
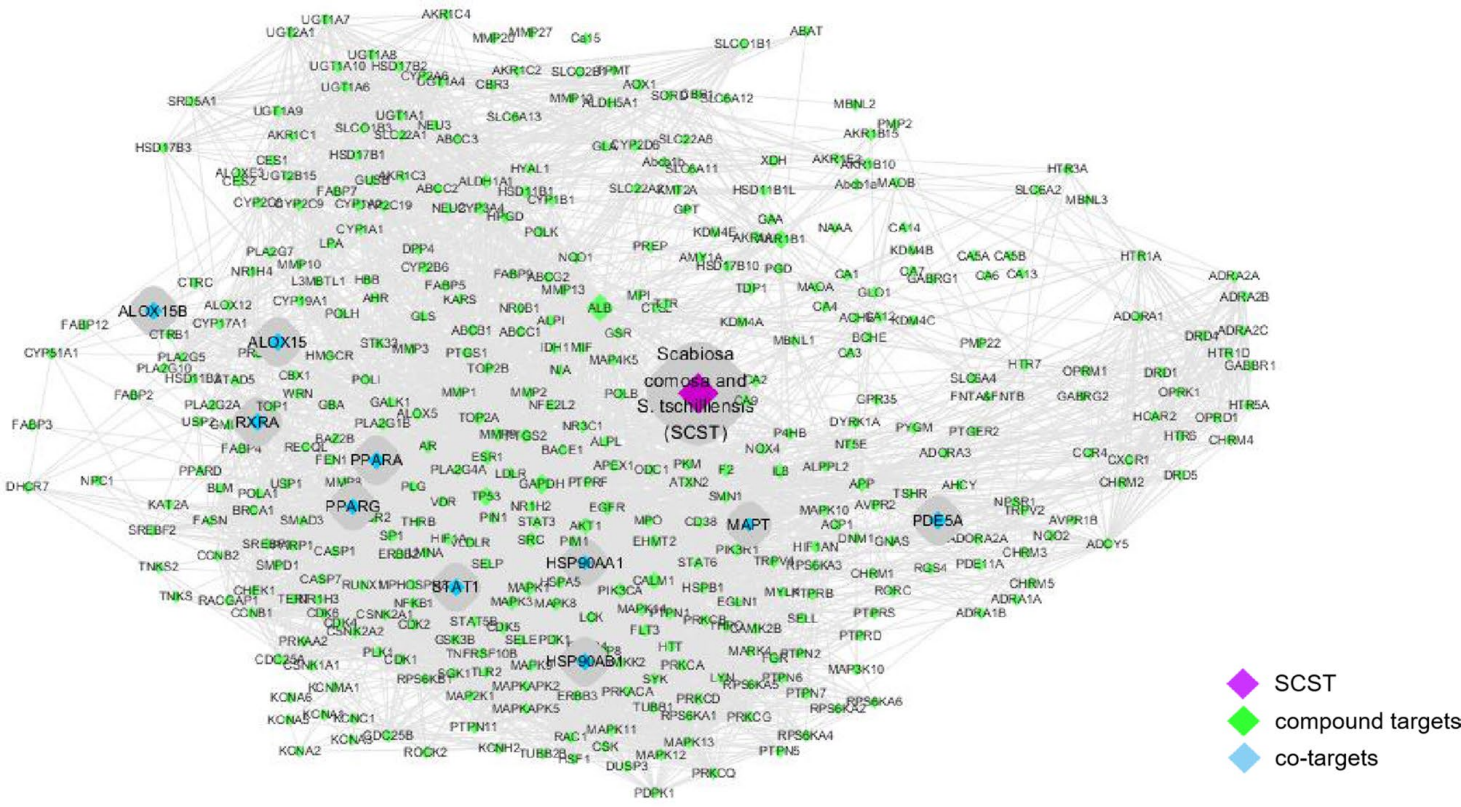
The network of SCST "compounds-targets-interacting proteins". The purple nodes represent SCST, the green nodes represent compound targets, and the blue nodes represent co-targets.

Network pharmacology is a powerful tool for studying the medicinal properties of complex natural products. According to the filters previously described, a total of 73 SCST components were selected as candidates for further investigation, including 27 flavonoids, 11 phenylpropanoids, 18 terpenoids, 4 iridoids, 4 coumarins, 3 aromatic acids, 2 steroidal acids and 4 other compounds. We obtained 403 candidate targets that were targeted by the above 73 components (total frequency of 1723). By mapping the candidate targets to the 66 known genes associated with hepatic fibrosis obtained from OMIM and DrugBank, 10 co-targets were found, including HSP90AA1, PPARG, MAPT, HSP90AB1, STAT1, and PPARA. Based on these results, the network of SCST “compound-target-interacting proteins” was constructed using Cytoscape 3.5.2 software, as shown in Fig. 1.

After analysing the topological characteristics, if the *degree*, *betweenness*, and *closeness* of the node were greater than the median of the corresponding parameters, the node was designated a hub node. The results showed that the hub nodes included 15 key compounds and 8 co-targets, and the detailed information is listed in Supplementary Tables S3 and S4, respectively.

These 15 key compounds included 9 flavonoids and 5 phenylpropanoids, and the top five compounds in the *closeness* order are quercetin, apigenin, luteolin, caffeic acid, and quercetin-3-glucoside; therefore, we preliminarily believe that the flavonoids and phenylpropanoids of SCST are important ingredients against hepatic fibrosis.

The co-targets are closely related to liver diseases, and in the *degree* order, the top four included heat shock protein 90AA1 (HSP90AA1), peroxisome proliferator-activated receptor G (PPARG), heat shock protein 90AB1 (HSP90AB1), and signal transduction and transcriptional activator 1 (STAT1). STAT has 6 family members, STAT1-STAT6. Among them, STAT1 is a negative regulator of liver fibrosis, which can inhibit the activation and proliferation of HSCs and promote the killing ability of NK on activated HSCs^40^. PPARG, which is a member of the PPAR family, is mainly expressed in adipose tissue and the liver. By regulating the expression of related genes, PPARG can correct liver lipid metabolism disorders, reduce oxidative stress, reduce the fibrotic cytokine α-smooth muscle actin (α-SMA) in HSCs and is essential for both adipocyte differentiation and HSC quiescence^41–43^. Heat shock proteins (HSPs) are also known as stress proteins and are divided into the HSP70 family, HSP90 family, HSP100 family, and some small heat shock proteins. Among them, HSP90 is a class of molecular chaperone proteins. Some studies have found that HSP90 inhibitors can induce HSC apoptosis through neurophospholipase or NF-κB depending on the mechanism^44^. Therefore, we selected HSP90AA1, PPAR and STAT1 as the final targets to verify the hepatoprotective effects of SCST in preparation for subsequent mechanistic research.

**Figure 2:**
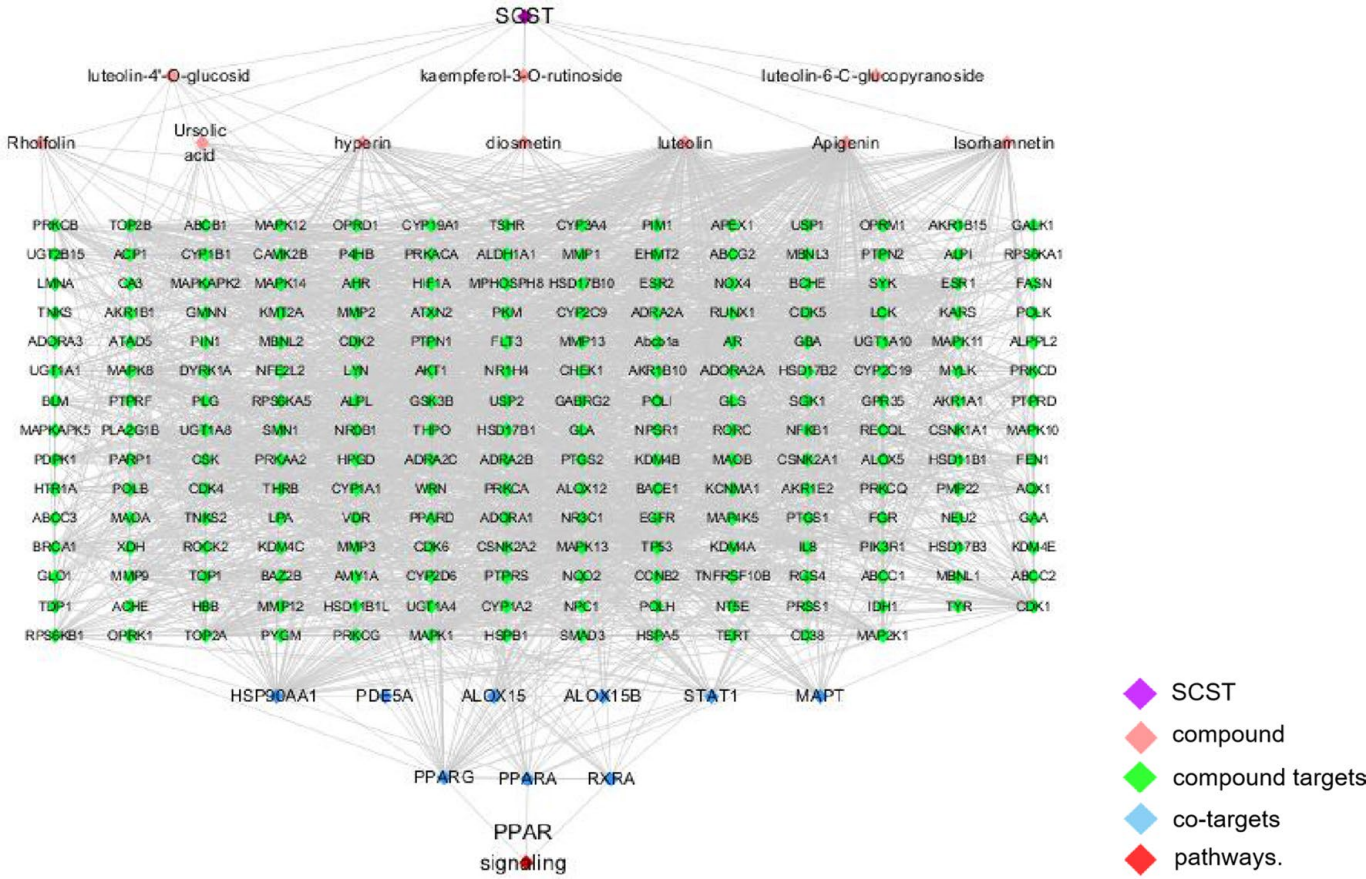
The “compound-target-pathway” network model. Purple nodes represent herbs, pink nodes represent SCST compounds, green nodes represent SCST compound targets, blue nodes represent co-targets, and red nodes represent pathways.

Furthermore, based on enrichment analysis of key co-targets, the PPAR signalling pathway^45^ was most correlated with anti-hepatic fibrosis in SCST (*P* = 0.00364). Related studies have shown that the PPAR pathway is involved in adipogenesis, lipid metabolism, inflammation and the maintenance of metabolic homeostasis. The effects of PPARγ agonism on the liver remain under debate, with some studies showing that it promotes hepatic steatosis through upregulation of the genes involved in lipid uptake and storage and other studies showing that it prevents hepatic steatosis and fibrosis^46^. The "compound-target-pathway" network model is shown in Fig. 2.

### Molecular docking

**Figure 3:**
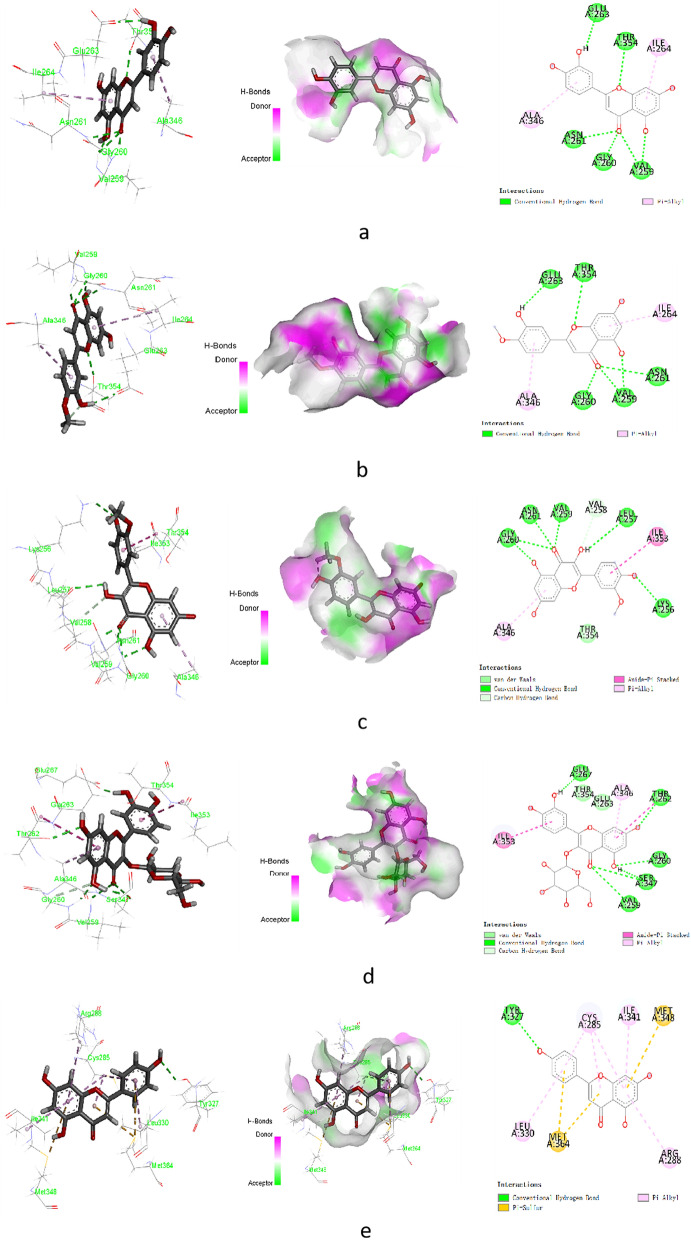
The molecular docking results between the compounds (**a**) luteolin, (**b**) diosmetin, (**c**) isorhamnetin, and (**d**) hyperin and the protein XDH. (**e**) The molecular docking results between the compounds apigenin and the protein PPARG.

Analysing the above Fig. 2 and combining the network topology parameters, it can be found that PPARG is the key target in the PPARG signalling pathway, and the corresponding compound is apigenin. Although STAT1 and HSP90AA1 have no direct targets, SCST indirectly affects PPARG, STAT1 and HSP90AA1 to exert anti-fibrosis effects through XDH, which is also closely related to liver fibrosis. The PDB codes of the above key targets (PPARG (3vso), XDH (3uni)) were searched for and sorted out from the RCSB PDB website. Schrodinger molecular docking technology is used for docking, and the interaction between these targets, the surrounding key amino acids and the binding of their target proteins at the active site are shown in Fig. 3. The docking result energy values of the compounds are listed in Table 1.

**Table 1:** Docking score results of the Schrodinger docking compound.

Compound	Target	PDB	Docking score
Luteolin	XDH	3uni	− 7.813
Diosmetin	XDH	3uni	− 7.479
Isorhamnetin	XDH	3uni	− 6.053
Hyperin	XDH	3uni	− 6.406
Apigenin	PPARG	3vso	− 5.805

The molecular docking results showed that the five compounds docked well with their corresponding proteins. The surrounding key amino acids mainly played a role in the formation of hydrogen bonds, aromatic bonds and hydrophobic bonds and the co-crystallized surrounding amino acids within the receptor protein itself (RCSB Protein Data Bank website) were basically the same; combined with Table 1, the docking energy values are relatively small, indicating that the compound can stably bind to the receptor protein and play a role.

### Upregulation of the pro-fibrotic marker α-SMA and the reduction in the fat-storing phenotype in activated LX-2 cells

**Figure 4:**
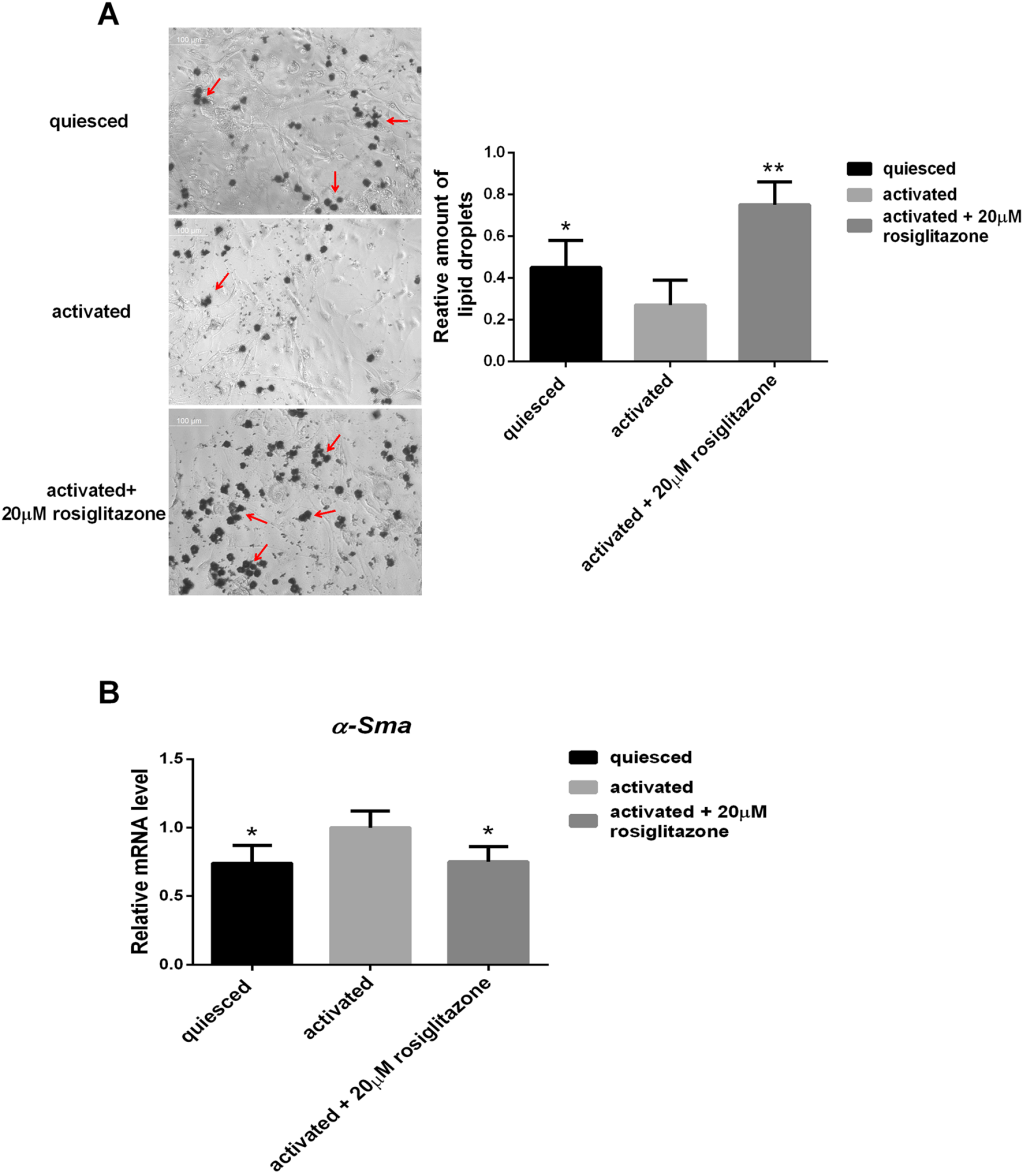
Lipid droplets and the mRNA expression levels of the fibrotic marker α-SMA in LX-2 cells. (**A**) Lipid droplets in LX-2 cells were stained by Oil Red O and quantitatively detected. Oil red O staining in activated LX-2 cells significantly decreased, while staining markedly increased after the addition of 20 µM of the PPARγ agonist rosiglitazone (*n* = 3). Arrows refer to the stained lipid droplets. (**B**) Compared with quiesced cells, the mRNA level of α-SMA in activated LX-2 cells increased, while the *α-Sma* gene was conspicuously suppressed by 24 h of treatment with 20 µM rosiglitazone (*n* = 3). Mean values ± SD are presented. ^##^*P* < 0.01 *vs*. the activated LX-2 group, ^#^*P* < 0.05 *vs*. the activated LX-2 group.

In order to identify the activated phenotype of LX-2, the mRNA level of the pro-fibrotic marker α-SMA and the presence of cytoplasmic lipid droplets were analysed by a qRT-PCR assay and Oil Red O staining, respectively. Compared with quiesced LX-2 cells cultured with 2% FBS, upregulated *α-Sma* and decreased oil red O staining were found in activated LX-2 cells cultured with 10% FBS. In addition, treatment with 20 µM of the PPARγ agonist rosiglitazone markedly suppressed *α-Sma* expression and increased oil red O staining (Fig. 4).

### The anti-proliferative effects of the flavonoids in SCST on LX-2 cells

**Figure 5:**
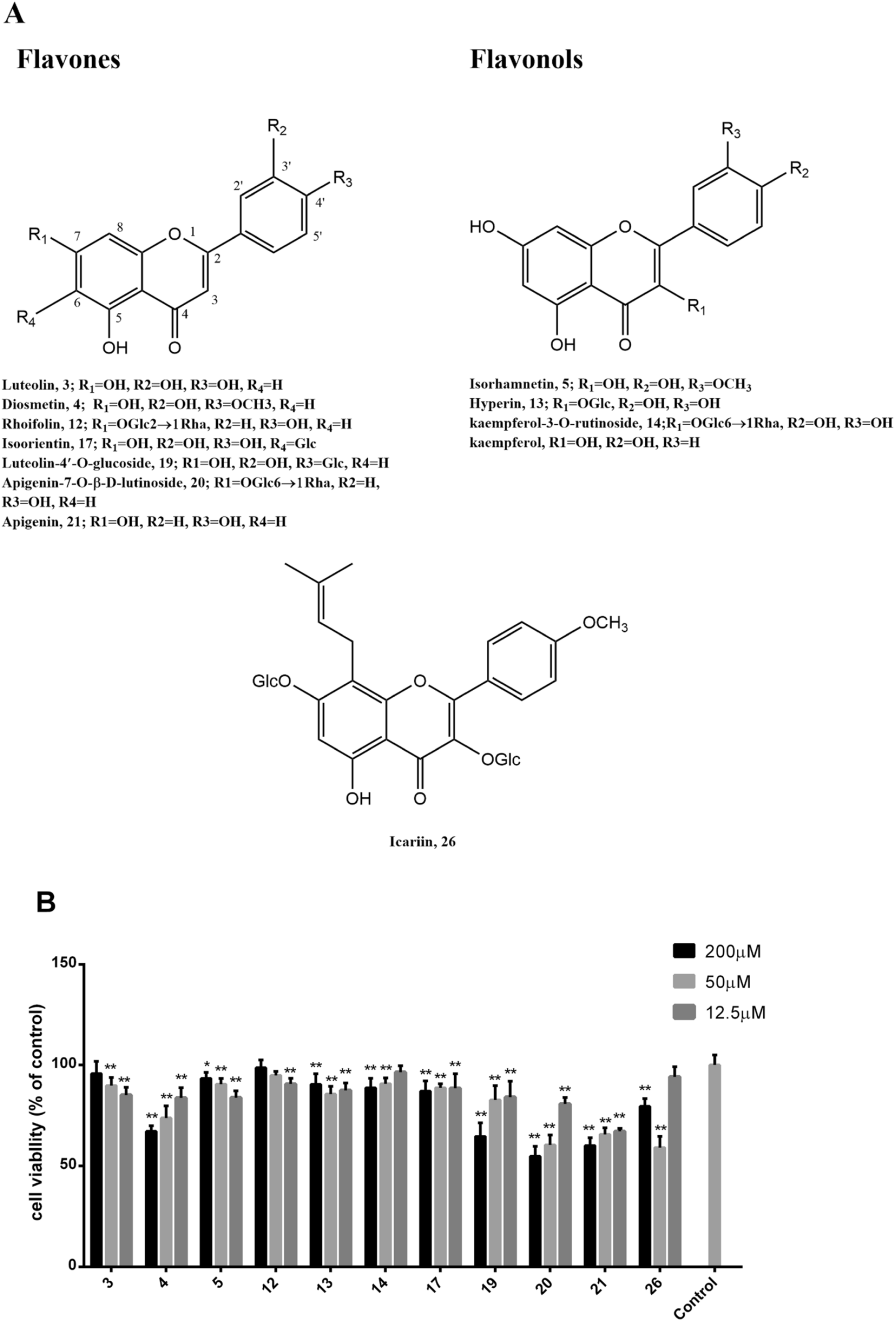
The structure of the flavonoids found in SCST. (**A**) The anti-proliferative activity of flavonoids found in SCST in LX-2 cells were tested by MTT assay. (**B**) The anti-proliferative activity of the flavonoids found in SCST in LX-2 cells as tested by the MTT assay (*n* = 5). Mean values ± SD are presented. **P < 0.01 versus the control group, *P < 0.05 versus the control group.

To evaluate the anti-hepatic fibrosis of SCST, 36 ingredients collected from SCST, including 13 predicted key compounds based on the network pharmacology analysis, were investigated for their anti-proliferation effects on LX-2 cells with the MTT assay. The chemical structures and results of the flavonoids in SCST are shown in Fig. 5, and the results of the other compounds are shown in Supplementary Fig. S1. Of the 37 compounds tested, 18 exhibited anti-proliferative activity in LX-2 cells in the concentration range of 12.5–200 μM, in which eleven compounds were flavonoids (**3, 4, 5, 12, 13, 14, 17, 19, 20, 21, 26)** (Fig. 5), accounting for 61% of the total compounds, two were terpenoids, corosolic acid (**30**) and ursolic acid (**33**), which displayed the strongest anti-proliferative activity, and others were iridoids, steroids, etc. Furthermore, we found that the presence of a single C_4_′–OH in the flavonoids could be the most important feature for the anti-proliferative effects; for example, apigenin (**21**) showed the strongest anti-proliferative activity among all flavonoids. In addition, the presence of a C_3_–OH, C_3_′–OH, or C_4_′–OCH_3_ group produced some negative effects on the activity, for example, diosmetin (**4**), isorhamnetin (**5**), diosmetin (**4**) and apigenin (**21**). The C_3_′–OH group could have the greatest interference on the activity; for example, luteolin (**3**) and **21**. Interestingly, most of the glucose groups had no contribution to activity, but the glucose group at C_4_′ increased the anti-proliferative activity when a C3′–OH was present; for example, in luteolin (**3**) and luteolin-4′-O-glucoside (**19)**.

It is worth noting that the seven phenylpropanoids of SCST, including 1,5-dicaffeoylquinic acid (**9**), isochlorogenic acid C (**10**), isochlorogenic acid B (**11**), chlorogenic acid (**22**), isochlorogenic acid A (**24**), neochlorogenic acid (**29**) and caffeic acid (**36**), exhibited significant pro-proliferative activity in the concentration range of 12.5–400 μM in LX-2 cells, and these compounds are derivatives of one or two chlorogenic acids bound to caffeic acid at different positions. Except for **9**, their pro-proliferative activity was positively correlated with their concentration, and the chemical structures and results of these SCST phenylpropanoids are shown in Supplementary Fig. S2.

Combined with the results of network pharmacology, we think that these flavonoid derivatives with similar nuclear structures may be responsible for most of the anti-proliferative activity of SCST. Therefore, we further explored the relationship between the flavonoids of SCST and the key targets predicted via network pharmacology analysis.

### The effects of the flavonoids found in SCST on the expression of key targets

To verify the results of the network analysis and explore the structure–activity relationship of the flavonoids in SCST, the gene expression of the key co-targets HSP90AA1, PPARG and STAT1 in each group was detected by qRT-PCR. Moreover, based on a previous screening of SCST blood components, kaempferol (Fig. 4A), a metabolite of isorhamnetin (**5**) and kaempferol-3-O-rutinoside (**14**), was added for further study^9^, and icariin (**26**) (Fig. 4A), whose structure is less similar to those of the other SCST flavonoids, was removed. The results showed that treatment with flavonoids **3, 4, 5, 12**, **13**, **14**, **17**, **19**, **20**, **21** and kaempferol in the concentration range of 12.5–50 μM with 20 µM of the PPARγ agonist rosiglitazone for 24 h resulted in changes in the expression of the key co-targets to different degrees compared with the control group (activated LX-2 cells) (Fig. 5).

**Figure 6:**
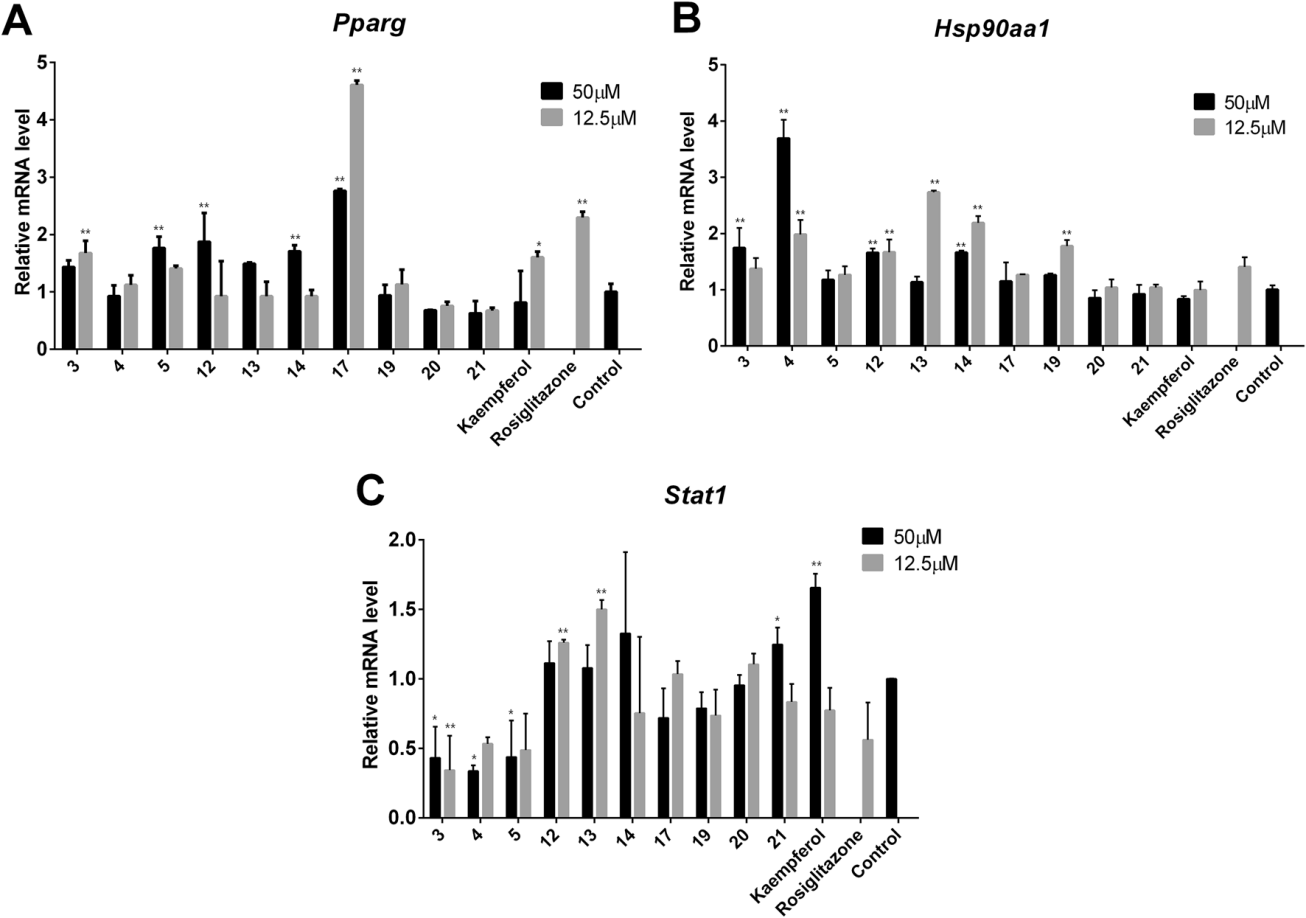
The mRNA levels of the key co-targets HSP90AA1, PPARG and STAT1 were adjusted by the flavonoids of SCST. (**A**–**C**) The relative mRNA levels of *Pparg, Hsp90aa1* and *Stat1* in each intervention group (*n* = 3). Mean values ± SD are presented. **P < 0.01 *vs*. the control group, *P < 0.05 *vs*. the control group.

As shown in Fig. 6, the compounds isorhamnetin **(5),** rhoifolin **(12),** kaempferol-3-O-rutinoside (**14**), and isoorientin (**17)** at a concentration of 50 μM and luteolin (3)**,** isoorientin (**17)** and kaempferol at 12.5 μM effectively promoted the expression of *Pparg*, with the effect of isoorientin (**17)** being the most prominent. Second, luteolin (3)**,** diosmetin (**4**), rhoifolin **(12)** and kaempferol-3-O-rutinoside (**14**) at a concentration of 50 μM and diosmetin (**4**), rhoifolin **(12),** hyperin **(13)** and kaempferol-3-*O*-rutinoside (**14**) at 12.5 μM effectively promoted the expression of *Hsp90aa1*, with 12.5 μM diosmetin (**4**) being the most effective. Moreover, 50 μM kaempferol significantly increased the expression of the *Stat1 gene* by approximately 70%*,* whereas luteolin (**3**)**,** diosmetin (**4**), and isorhamnetin **(5)** dramatically reduced the expression of the *Stat1 gene* and showed the opposite profile. Treatment with 20 µM rosiglitazone resulted in the downregulation of PPARG but there were no pronounced effects on the *Hsp90aa1* and *Stat1* genes. The effects of many natural products on biological activity are multifaceted, and many natural products show activation at low concentrations and inhibition at high concentrations, or vice versa. Pparg and Stat1 play pivotal roles in the pathogenesis of liver fibrosis, but there is an upstream or downstream relationship between the two. For example, in this experiment, the effect of kaempferol on the upregulation of STAT1 is better at high concentrations than at low concentrations, while the downregulation of PPARG was better at high concentrations than at low concentrations.

Based on the above results, we found that the coexistence of C_3_′–OH and C_4_′–OH groups in these flavonoids could be the most important factor for upregulating the expression of the *Pparg* gene; if one of these moieties is lost or C_3_′-OH is replaced by a hydroxymethyl, the effects of compounds will be weakened; for example, in the cases of luteolin (**3**), apigenin (**21**)**,** and diosmetin (**4**) (*P* < 0.05). Compared with the coexistence of the two hydroxyl groups on the B ring, the presence of a rhamnoside at C_6_ further upregulated *Pparg*, for example, with isoorientin (**17)** and luteolin (**3**) (*P* < 0.01)**.** Moreover, the coexistence of C_4_′-OCH_3_ and C_3_′-OH in diosmetin (**4**) exhibited the optimum efficiency in upregulating the *Hsp90aa1* gene; if C_4_′-OCH_3_ was replaced by a hydroxyl, the effect of upregulating the *Hsp90aa1* gene would be weakened; for example, with luteolin (**3**). Furthermore, the presence of a C_3_-OH could play the most negative role of flavonoids in upregulating the *Hsp90aa1* gene; for example, with diosmetin (**4**) and isorhamnetin **(5)** at 50 μM (*P* < 0.05)**,** and a glucose group instead of a hydrogen at C_3_ could alleviate the negative effects from the C_3_–OH; for example, with kaempferol, hyperin **(13)**, and kaempferol-3-*O*-rutinoside (**14**) (*P* < 0.01 or *P* < 0.05). In contrast, the presence of a C_3_–OH and a single hydroxyl on the B ring could be the most important features of the flavonoids in upregulating the *Stat1* gene. For example, kaempferol showed the strongest stimulating activity to upregulate the *Stat1* gene among all flavonoids, and a glucose group instead of a hydrogen at C_3_ or the presence of alone at C_4_′-OH without a C_3_-OH would lead to a reduced ability to upregulate the *Stat1* gene; for example, with kaempferol, hyperin **(13),** and apigenin (**21**) (*P* < 0.01 or *P* < 0.05).

**Figure 7:**
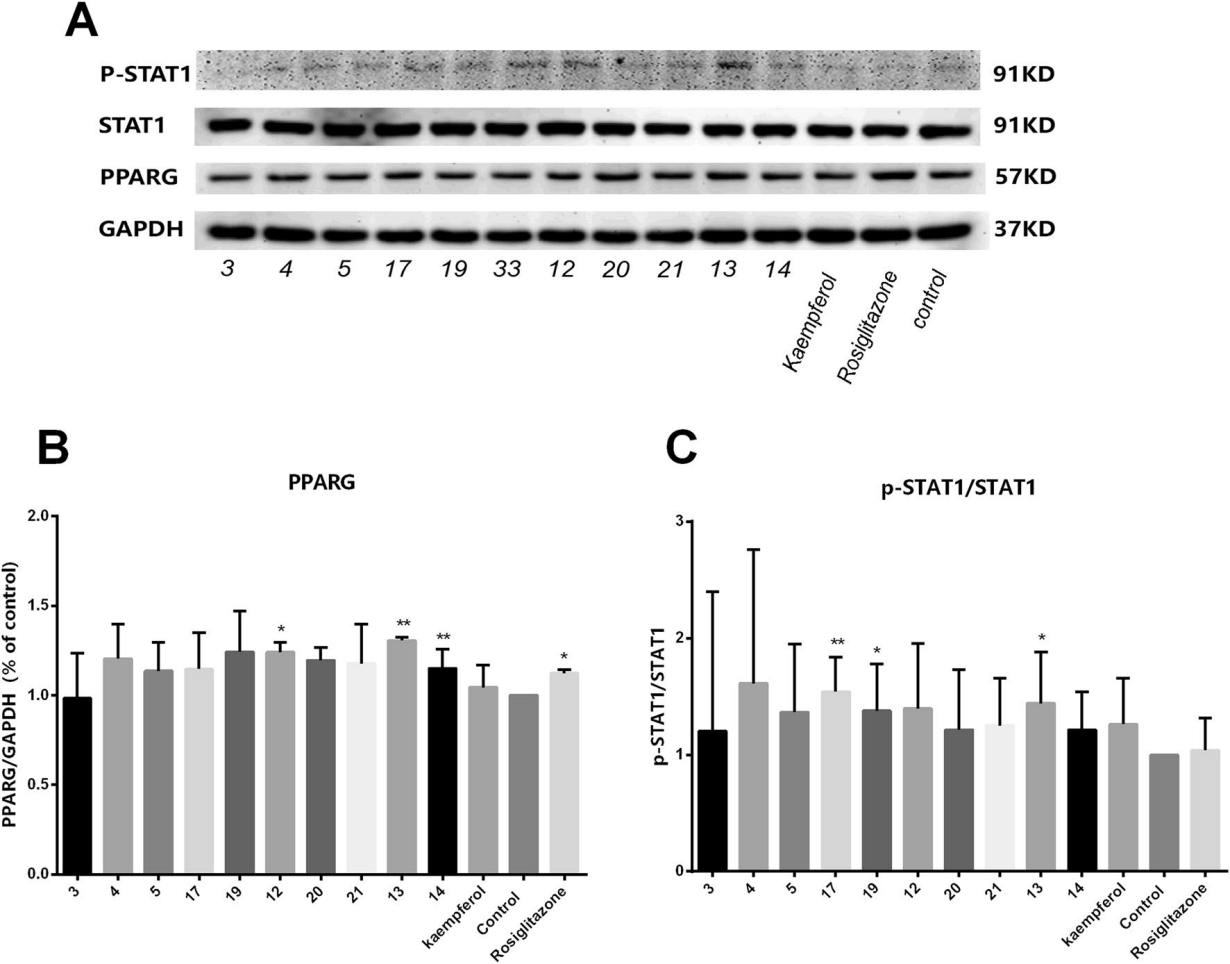
The flavonoids of SCST treated for 24 h at 50 µM altered the protein expression of PPARG, p-STAT1, and STAT1 in activated LX-2 cells. (**A**) Representative Western blots showing the effects of the flavonoids of SCST on the expression of PPARG, p-STAT1, and STAT1, with GAPDH used as the loading control. (**B**) The protein expression levels of PPARG in each group (*n* = 4). (**C**) The ratio of p-STAT1/STAT1 in each group (*n* = 3). Mean values ± SD are presented. **P < 0.01 *vs*. the control group, *P < 0.05 *vs*. the control group.

Furthermore, the protein levels of PPARG, STAT1 and p-STAT1 in each group treated with 50 μM flavonoids and rosiglitazone were analysed by Western blotting. As shown in Fig. 7, compared with the control group, 50 μM of each rhoifolin **(12),** hyperin **(13)**, and kaempferol-3-O-rutinoside (**14**) and 20 μM rosiglitazone effectively increased the expression of PPARG (*P* < 0.01 or *P* < 0.05). Activated STAT1 is tyrosine- and serine-phosphorylated. It then forms a homodimer and migrates into the nucleus to drive the expression of its target genes^47^. Treatment with 50 μM isoorientin (**17),** luteolin-4′-O-glucoside (**19**) or hyperin **(13)** significantly increased the ratio of p-STAT1/STAT1 (*P* < 0.01 or *P* < 0.05), and most of the flavonoids tested at 50 μM showed an upward trend in the expression of PPARG and the ratio of p-STAT1/STAT1.

## Conclusion

SCST has been used in Mongolian medicine to treat liver diseases, and the ingredients of SCST mainly consist of flavonoids, phenylpropanoids and iridoids. Accumulating data have demonstrated that different types of compounds in SCST seem to have distinct roles in treating liver diseases. It has been shown that the flavonoid-rich aqueous extract of SCST can attenuate liver fibrosis by selectively inhibiting Smad3 phosphorylation to downregulate the transcription of fibrotic genes in a rat model of CCl4-induced hepatic fibrosis or in TGF-β1-activated HSCs. Our early observations also suggest that the majority of constituents that migrate to the blood from the SCST ethanol extract are flavonoids and phenylpropanoids^9^. Most of the SCST flavonoids show anticancer activity in *vitro*^25^. In contrast, most of the SCST phenylpropanoids alleviate oxidative stress in H_2_O_2_-induced HL-7702 cells, an immortalized human liver line (this study has not been published). In addition, the plant extracts were demonstrated to have excellent anti-HCV activity, and the phenylpropanoids chlorogenic acid and 3,5-dicaffeoylquinic acid showed significant anti-HCV activity^20^.

To systematically explore the pharmacodynamic advantages of SCST on liver fibrosis, we employed network pharmacology combined with an experimental verification approach to identify the active ingredients and molecular mechanisms of SCST. Our results demonstrated that SCST could target HSP90AA1, PPARG, HSP90AB1, STAT1, etc., further regulating the PPARG signalling pathway that is closely related to liver fibrosis, and the flavonoids and phenylpropanoids of SCST are important ingredients against hepatic fibrosis. The proliferation and activation of HSCs is the key stage of hepatic fibrosis. In our results, all of the tested flavonoids exhibited anti-proliferative activity against HSCs, and in contrast, all but one of the phenylpropanoids displayed significant pro-proliferative activity. Ganbold and colleagues demonstrated that isorhamnetin and 3-*O*-methylquercetin had stronger inhibitory effects on the proliferation of rat hepatic stellate cells (HSCs-T6) and suggested that methyl groups at the C-3′ and C-3 positions may have functional roles in exerting biological activity^48^. He et al. also showed that chrysin, luteolin, apigenin, hesperetin and 3′,4′-dimethoxyhesperetin suppress hepatocyte apoptosis and apparently have hepato-protective effects against acute liver failure induced by lipopolysaccharide/d-galactosamine. In addition, they suggested that the C2-3 double bond on the A ring and a hydroxyl group at C3′ or C4′ on the B ring increase the protective activities; however, hydroxymethylation at C3′ and C4′ reverses the activity^49^. Our results showed that the presence of a flavonoid C4′-OH could be essential for the anti-proliferative effects, and C_3_-OH, C_3_′-OH, and C_4_′-OCH_3_ groups produced some negative effects on the activity of the flavonoids. In addition, apigenin had the optimal anti-proliferative effect, which is consistent with He’s report^50^.

The results of network pharmacology were verified by qRT-PCR and WB technology. This indicates that the majority of SCST flavonoids regulate the expression of the *Hsp90aa1*, *Pparg* and *Stat1* genes and show an upward trend in the expression of PPARG and the ratio of p-STAT1/STAT1. It appears that the relationship between the gene expression of *Stat1* and structure have the opposite trend of *Hsp90aa1* and *Pparg*. Collectively, these observations suggest that the anti-proliferative activity of these flavonoids in LX-2 cells could be associated with regulation of the expression of the *Hsp90aa1*, *Pparg* and *Stat1* genes and the PPARG, p-STAT1, and STAT1 proteins.

The bidirectionality or multanimity of traditional medicinal herbs has been debated^51^; for example, the haemostatic components in *Panax notoginseng* are notoginseng and quercetin, and the antithrombotic components are saponins^52^. The therapeutic or toxic effects of *Rheum palmatum L.* depend on the dosage level^53^. The present study demonstrates that flavonoids and phenylpropanoids in SCST are seemingly contradictory or synergistic, while the exact roles of the different types of compounds in SCST remain to be understood. Moreover, these results indicate that SCST has multi-targeted and multi-component anti-hepatic fibrosis synergistic effects.

## Supplementary Information

Supplementary Information 1.
