# Supplementary material for: Systematic profiling of the effective ingredients and mechanism of Scabiosa comosa and S. tschilliensis against hepatic fibrosis combined with network pharmacology
Source: Sci Rep. 2021 Jan 28;11:2600. doi: 10.1038/s41598-021-81399-x (PMC7843997; doi:10.1038/s41598-021-81399-x)
Supplement: Supplementary file 1 — Supplementary Information 1. [file 41598_2021_81399_MOESM1_ESM.pdf]

Systematic profiling of the effective ingredients and mechanism of Scabiosa comosa and S. tschilliensis against hepatic fibrosis combined with network pharmacology

Qianwen Chen[1]& Yuanyuan Wang[1]& Feixiang Ma[1]& Mengdi Han[1]& Zhen Wang[1]& Peifeng Xue[1,*]& Jingkun Lu[2,*]

(1. Department of Basic Medicine, Medical University of Inner Mongolia, Jinshan Development Zone, Hohhot, Inner Mongolia, China. 2. Department of Pharmacy, Medical University of Inner Mongolia, Jinshan Development Zone, Hohhot, Inner Mongolia, China)

Corresponding authors detail:

1. Peifeng Xue, email adress: xpfdc@vip.sina.com.
2. Jingkun Lu, email adress: 360569392@qq.com.

Gels/blots are used in figures：

p-STAT1：91KD

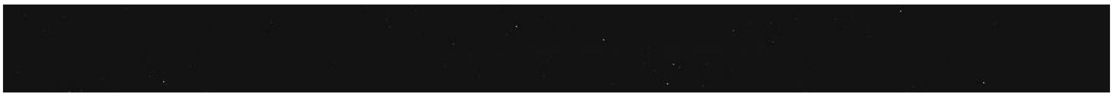

p-STAT1(higher-contrast image)：91KD

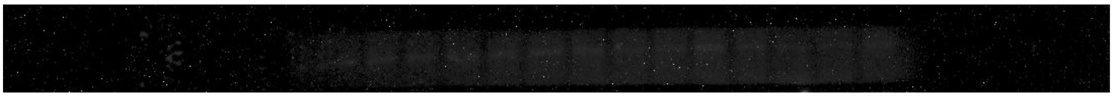

STAT1：91KD

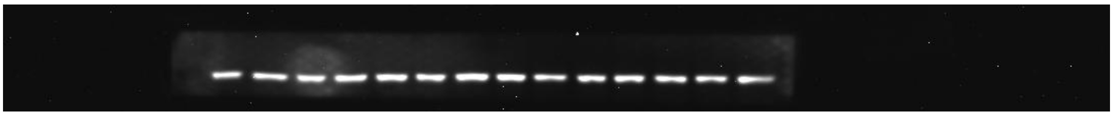

PPARG:57KD

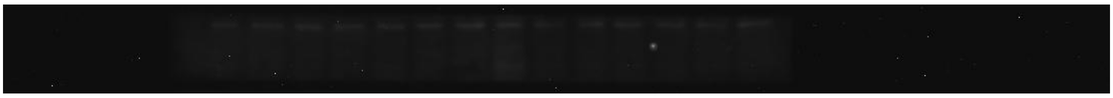

GAPDH:37KD

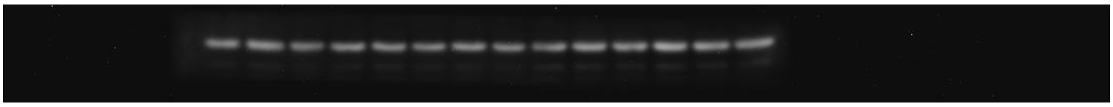

Supplementary Figure S1:

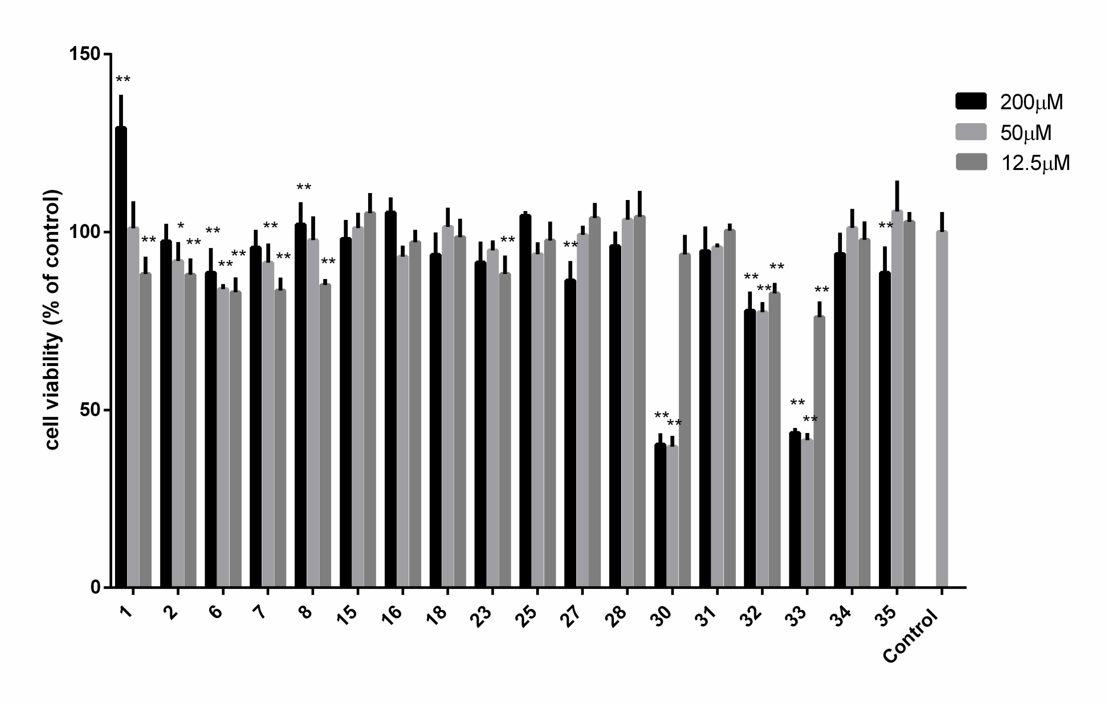

Supplementary Figure S1. The anti-proliferative activity of flavonoids found in SCST in LX-2 cells were tested by MTT assay.(B) The anti-proliferative activity of the flavonoids found in SCST in LX-2 cells as tested by the MTT assay (*n*=5). Mean values ± SD are presented. ***P* < 0.01 versus the control group, **P* < 0.05 versus the control group.

Supplementary Figure S2A:

A

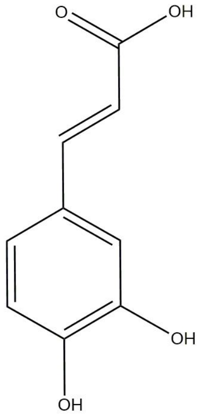

Caffeic acid, 37

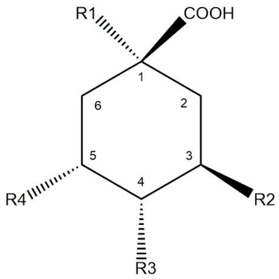

1,5-Dicaffeoylquinic acid, **9**, R1=37, R2=OH, R3=OH, R4=37;
Isochlorogenic acid C, **10**, R1=OH, R2=OH, R3=37, R4=37;
Isochlorogenic acid B, **11**, R1=OH, R2=37, R3=37, R4=OH;
Chlorogenic acid, **22**, R1=OH, R2=37,R3=OH, R4=OH;
Isochlorogenic acid B, **24**, R1=OH, R2=37, R3=OH, R4=37;
Neochlorogenic acid, **29**, R1=OH, R2=OH, R3=OH, R4=37;

Supplementary Figure S2B:

B

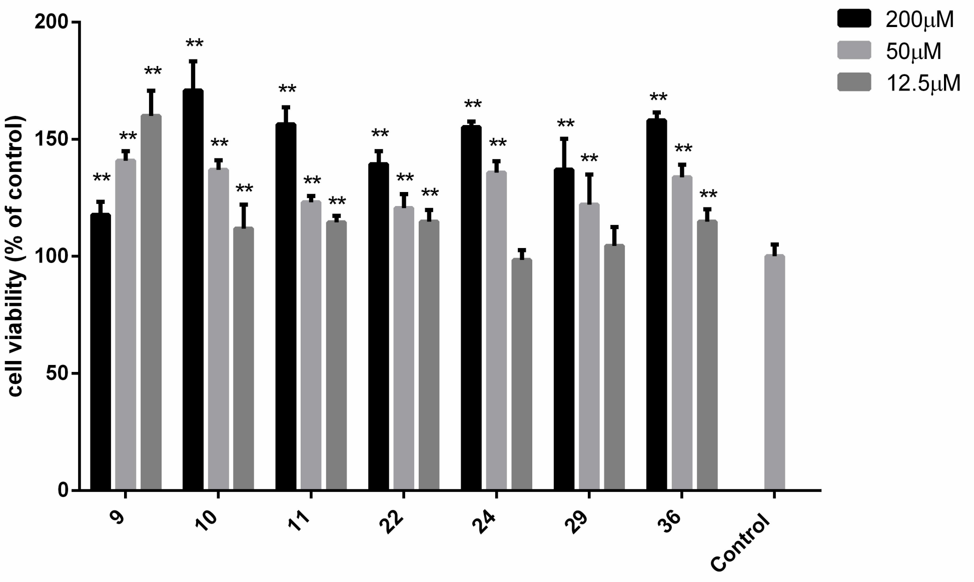

Supplementary Figure S2. The structure of the phenolic acids found in SCST. (A) The anti-proliferative activity of phenolic acids found in SCST in LX-2 cells were tested by MTT assay.(B) The anti-proliferative activity of the flavonoids found in SCST in LX-2 cells as tested by the MTT assay (*n*=5). Mean values ± SD are presented. ***P* < 0.01 versus the control group, **P* < 0.05 versus the control group.

Supplementary Table S1: The information of 73 screened compounds of STCT

Codes	Compound	MolecularFormula	CAS	Kinds	OB	DL	references
1	Apigenin	$C_{15}H_{10}O_5$	520-36-5	flavonoids	23.06	0.21	7, 8, 20, 21, 22, 24
2	Luteolin	C15H10O6	491-70-3	flavonoids	36.16	0.25	7, 8, 20, 21, 22, 24
3	Diosmetin	C16H12O6	520-34-3	flavonoids	31.14	0.27	22, 2
4	Quercetin	C15H10O7	117-39-5	flavonoids	46.43	0.28	21, 22, 24
5	Isorhamnetin	C16H12O7	480-19-3	flavonoids	49.60	0.31	21, 22, 24
6	Epicatechin	C15H14O6	35323-91-2	flavonoids	48.96	0.24	21
7	Apigetrin (Apigenin-7-O-glucoside)	C21H20O10	578-74-5	flavonoids	9.68	0.74	7, 9, 20
8	Apigenin-4'-O-β-glucoside	C21H20O10	20486-34-4	flavonoids	N/A	N/A	20,25
9	Swertisin	C22H22O10	6991-10-2	flavonoids	31.83	0.75	22
10	Apigenin-7-O-arabino(1-6)-β-glucoside	C26H28O14	N/A	flavonoids	N/A	N/A	9, 20
11	Apigenin-7-O-β-D-lutinoside	C26H30O15	N/A	flavonoids	N/A	N/A	7, 9
12	Rhoifolin	C27H30O14	17306-46-6	flavonoids	6.68	0.77	7, 9, 21, 22, 24
13	Rutin (kaempferol-3-O-rutinoside)	C27H30O16	153-18-4	flavonoids	3.20	0.68	22, 27
14	Kaempferol-3-o-β-D-6-O-(-p-hydroxycinnamoyl)-glucopyranoside	C30H26O13	N/A	flavonoids	N/A	N/A	7, 8, 22
15	Kaempferol-3-o-β-D-[3，6-di-p-hydroxycinnamoyl]-glucopyranoside	C39H32O15	N/A	flavonoids	N/A	N/A	7, 8, 25
16	Luteolin-7-O-β-glycoside	C21H20O11	5373-11-5	flavonoids	N/A	N/A	7, 9, 22

17	Luteolin-4'-O-glucosid	C21H20O11	N/A	flavonoids	N/A	N/A	7, 9, 22
18	Isoorientin (luteolin-6-C-glucopyranoside)	C21H20O11	4261-42-1	flavonoids	23.30	0.76	7, 20, 22
19	Luteolin-7-O-rutinoside	C27H30O16	N/A	flavonoids	N/A	N/A	9, 22
20	Hyperin	C21H20O12	482-36-0	flavonoids	6.94	0.77	23,28
21	Quercitrin	C21H20O11	522-12-3	flavonoids	4.04	0.74	21,29
22	Isoquercitrin (Quercetin-3-glucoside)	C21H20O12	482-35-9	flavonoids	1.86	0.77	20,22,30
23	Quercimeritrin	C21H20O12	491-50-9	flavonoids	2.85	0.79	22
24	Quercetin-6-glucoside	C21H20O12	N/A	flavonoids	16.75	0.80	22
25	Quercetin-3-rutinoside	C27H30O16	N/A	flavonoids	3.20	0.68	20
26	Icariin	C33H40O15	489-32-7	flavonoids	N/A	N/A	21,31
27	Tschilliensisin	C44H42O23	N/A	flavonoids	N/A	N/A	7
28	P-Coumaric acid	C9H8O3	501-98-4	phenylpropanoids	43.29	0.04	20,32
29	Caffeic acid	C9H8O4	331-39-5	phenylpropanoids	54.97	0.05	7, 9, 20, 21, 23
30	Caffeic acid methyl ester	C10H10O4	N/A	phenylpropanoids	N/A	N/A	7, 8
31	Ferulic acid	C10H10O4	1135-24-6	phenylpropanoids	39.56	0.06	9, 21
32	Chlorogenic acid	C16H18O9	327-97-9	phenylpropanoids	11.93	0.33	7, 9,20, 21, 22, 24
33	Neochlorogenic acid	C16H18O9	906-33-2	phenylpropanoids	11.93	0.33	7, 9, 20, 21, 24
34	1,5-Dicaffeoylquinic acid	C25H24O12	N/A	phenylpropanoids	N/A	N/A	9, 22
35	Isochlorogenic acid C (4,5-di-O-caffeoylquinic acid)	C25H24O12	32451-88-0	phenylpropanoids	1.78	0.69	9, 20, 22
36	Isochlorogenic acid B (3,4-dicaffeoylquinic	C25H24O12	14534-61-3	phenylpropanoids	1.78	0.69	9, 20

	acids)						
37	Isochlorogenic acid A (3,5-dicaffeoylquinic acids)	C25H24O12	2450-53-5	phenylpropanoids	1.79	0.69	9, 20
38	3,5-dicaffeoylquinic ethyl ester	C27H28O14	N/A	phenylpropanoids	N/A	N/A	9, 20
39	Sweroside	C16H22O9	14215-86-2	iridoid	N/A	N/A	8
40	Loganin	C17H26O10	18524-94-2	iridoid	5.90	0.44	7, 8, 25
41	Sylvestrosides I	C33H48O19	71431-22-6	iridoid	N/A	N/A	7, 8, 25
42	Sylvestrosides II	C35H50O20	71431-22-7	iridoid	N/A	N/A	7, 8, 25
43	P-hydroxybenzoic	C14H12O3	N/A	Aromatic acid	30.15	0.03	7, 8, 9
44	Protocatechuic acid	C7H6O4	99-50-3	Aromatic acid	N/A	N/A	20
45	Vanillic acid	C8H8O4	121-34-6	Aromatic acid	35.47	0.04	21， 33
46	Umbelliferone	C9H6O3	93-35-6	coumarin	27.37	0.05	24
47	Esculetin	C9H6O4	305-01-1	coumarin	22.97	0.07	9, 22
48	Bergaten	C12H8O4	484-20-8	coumarin	42.21	0.13	7, 20, 21
49	Umbellipronin	C24H30O3	23838-17-7	coumarin	N/A	N/A	7, 20, 21
50	Urceolide	C21H34O11	N/A	terpenoid	N/A	N/A	8
51	Ursolic acid	C30H48O3	77-52-1	terpenoid	16.77	0.75	7, 9, 20, 21, 24
52	Oleanolic acid	C30H48O3	508-02-1	terpenoid	29.02	0.76	7, 8
53	3β-23-dihydroxyursan-12-en-28-oic acid	C30H48O3	N/A	terpenoid	N/A	N/A	22, 26
54	Corosolic acid	C30H48O4	4547-27-4	terpenoid	N/A	N/A	20,34
55	scabiosaponins A	C63H102O31	N/A	terpenoid	N/A	N/A	20,34
56	scabiosaponins B	C68H110O35	N/A	terpenoid	N/A	N/A	20,34
57	scabiosaponins C	C69H112O36	N/A	terpenoid	N/A	N/A	20,34

58	scabiosaponins D	C58H94O27	N/A	terpenoid	N/A	N/A	20,34
59	scabiosaponins E	C57H92O26	N/A	terpenoid	N/A	N/A	20,34
60	scabiosaponins F	C58H94O27	N/A	terpenoid	N/A	N/A	20,34
61	scabiosaponins G	C64H104O32	N/A	terpenoid	N/A	N/A	20,34
62	scabiosaponins H	C63H102O33	N/A	terpenoid	N/A	N/A	20,34
63	scabiosaponins I	C57H92O28	N/A	terpenoid	N/A	N/A	20,34
64	scabiosaponins J	C57H92O28	N/A	terpenoid	N/A	N/A	20,34
65	scabiosaponins K	C62H92O28	N/A	terpenoid	N/A	N/A	20,34
66	hookerosides A		N/A	terpenoid	N/A	N/A	20,34
67	hookerosides B		N/A	terpenoid	N/A	N/A	20,34
68	β-sitosterol	C29H50O	83-46-5	steroids	N/A	N/A	8, 20,26, 35
69	Daucosterol	C35H60O6	474-58-8	steroids	20.63	0.63	7, 8
70	Adenosine	C10H13N5O4	58-61-7	others	15.98	0.18	7, 8, 25
71	Hentriacontane	C31H64	630-04-6	others	8.07	0.51	22, 36
72	Stearic acid	C18H36O2	57-11-4	others	17.83	0.14	8, 37
73	quinic acid	C7H12O6	77-95-2	others	63.53	0.06	20,22,38

Supplementary Table S 2: The detailed information of 36 tested compounds of SCST

Codes	Compound	Structure	CAS	Kinds	Screening basis	Key compounds
1	Protocatechuic acid	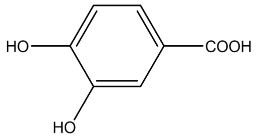	99-50-3	Aromatic acid	Linpinsk rules	NO
2	Isoquercitrin (Quercetin-3-glucoside)	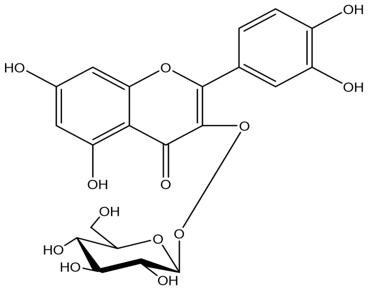	482-35-9	flavonoids	Anti-inflammatory	NO
3	Luteolin-7-O-β-glycoside	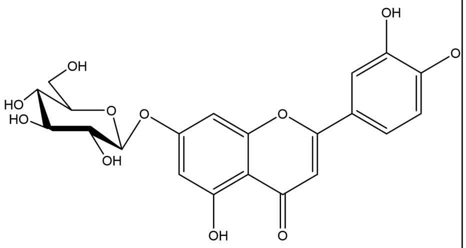	5373-11-5	flavonoids	Blood component	Yes
4	Diosmetin	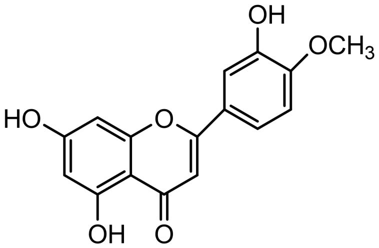	520-34-3	flavonoids	OB=31.14 DL=0.27	Yes
5	Isorhamnetin	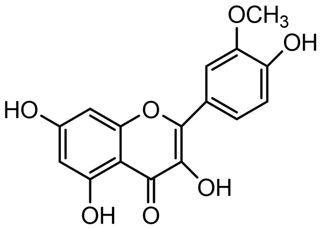	480-19-3	flavonoids	OB=49.60 DL=0.31	Yes
6	Loganin	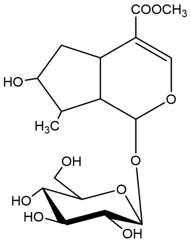	18524-94-2	iridoid	Anti-cancer	NO
7	P-Coumaric acid	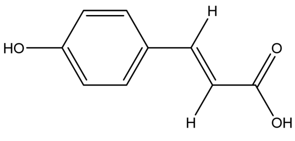	501-98-4	phenylpropanoids	anti-oxidation	NO

8	Quercetin	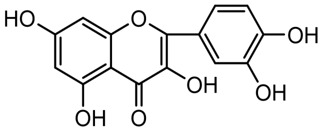	117-39-5	flavonoids	OB=46.43<br>DL=0.28	NO
9	1,5-Dicaffeoylquinic acid	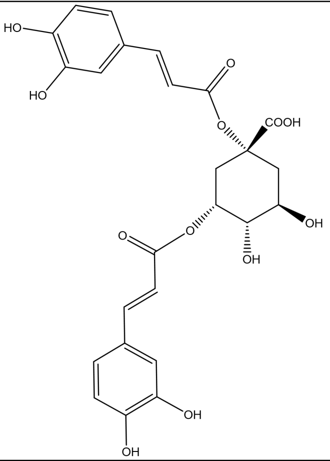	N/A	phenylpropanoids	Blood component	NO
10	Isochlorogenic acid C (4,5-di-O-caffeoylquinic acid)	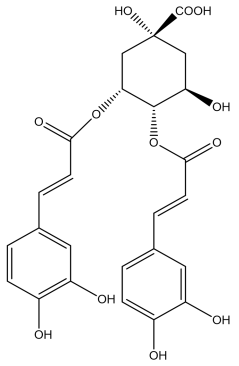	32451-88-0	phenylpropanoids	Blood component	NO
11	Isochlorogenic acid B (3,4-dicaffeoylquinic acids)	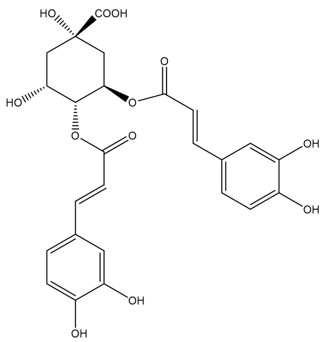	14534-61-3	phenylpropanoids	Blood component	NO
12	Rhoifolin	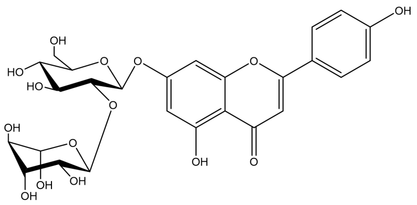	17306-46-6	flavonoids	Blood component	Yes
13	Hyperin	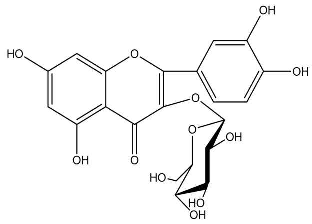	482-36-0	flavonoids	Anti-inflammatory	Yes

14	Rutin (kaempferol-3-O-rutinoside)	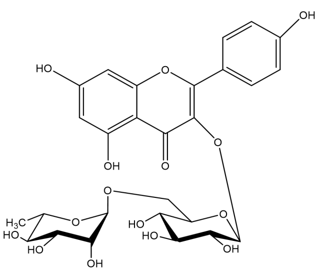	153-18-4	flavonoids	anti-oxidation	Yes
15	Adenosine	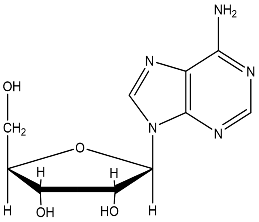	58-61-7	others	Anti-cancer	NO
16	Esculetin	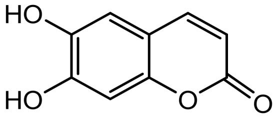	305-01-1	coumarin	Blood component	NO
17	Isoorientin (luteolin-6-C-glucopyranoside)	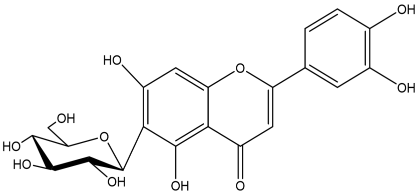	4261-42-1	flavonoids	OB=23.3 DL=0.76	Yes
18	quinic acid	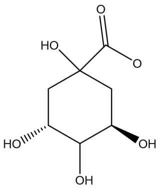	77-95-2	others	Linpinsk rules	NO
19	Luteolin-4'-O-glucosid	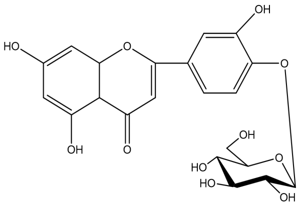	N/A	flavonoids	Blood component	Yes
20	Apigenin-7-O-β-D-lutinoside	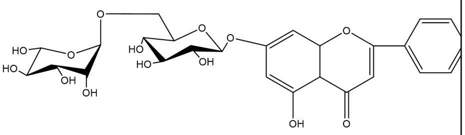	N/A	flavonoids	Apigenin Diglycosylation	Yes

21	Apigenin	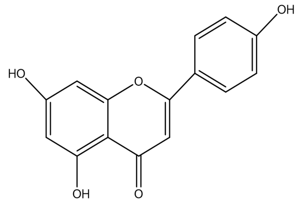	520-36-5	flavonoids	OB=23.06 DL=0.21	Yes
22	Chlorogenic acid	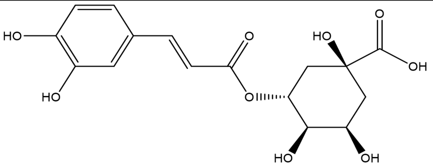	327-97-9	phenylpropanoids	Blood component	NO
23	Umbelliferone	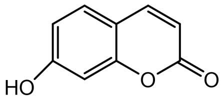	93-35-6	coumarin	Linpinsk rules	NO
24	Isochlorogenic acid A (3,5-dicaffeoylquinic acids)	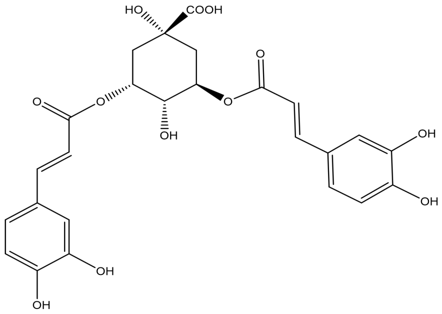	2450-53-5	phenylpropanoids	Blood component	NO
25	Oleanolic acid	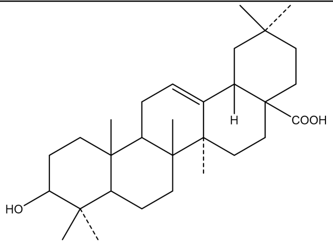	508-02-1	terpenoid	OB=29.02 DL=0.76	NO
26	Icariin	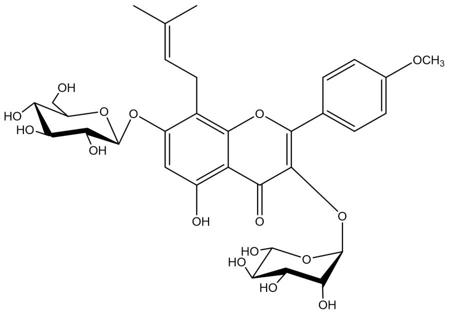	489-32-7	flavonoids	Antitumor	NO
27	Daucosterol	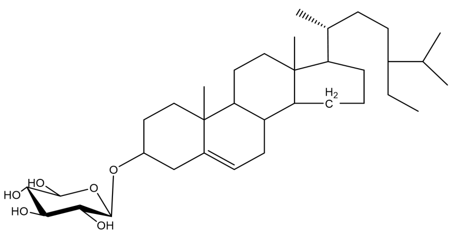	474-58-8	steroids	Linpinsk rules	NO

28	Luteolin -7-O-β-g lycoside	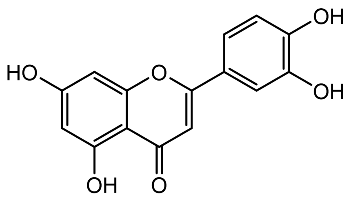	5373-11-5	flavonoids	Blood component	NO
29	Neochlorogenic acid	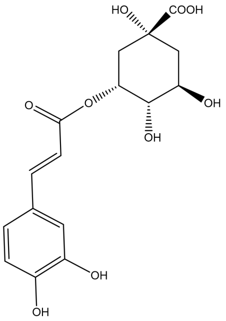	906-33-2	phenylpropanoids	Blood component	NO
30	Corosolic acid	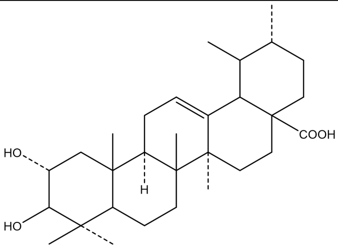	4547-27-4	terpenoid	Cure liver disease	NO
31	Quercitrin	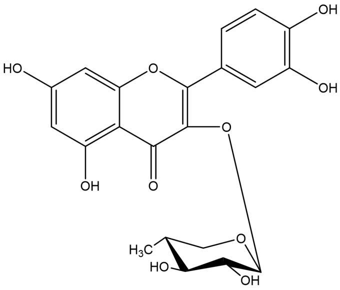	522-12-3	flavonoids	anti-oxidation	NO
32	β-sitosterol	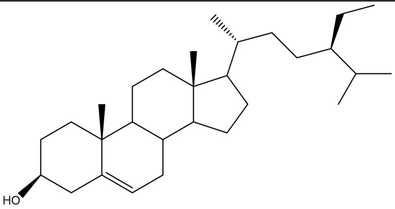	83-46-5	steroids	Anti-inflammatory	NO
33	Ursolic acid	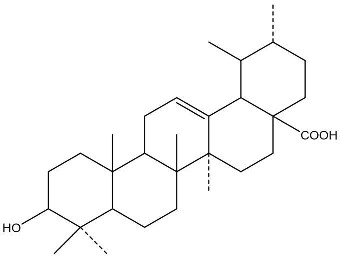	77-52-1	terpenoid	Blood component	NO
34	Stearic acid	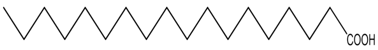	57-11-4	other	Antibacterial	NO
35	Caffeic acid methyl ester	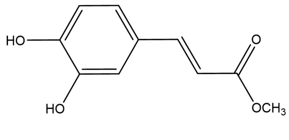	N/A	phenylpropanoids	Linpinsk rules	NO

36	Caffeic acid	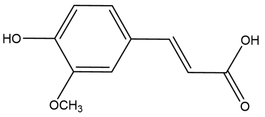	331-39-5	phenyl propanoids	Blood component	NO

Supplementary Table S3: The detailed information of 15 key compounds of STCT

Compound	Degree	Betweenness	Closeness
quercetin（8）	129	0.08897565	0.73972603
Apigenin（21）	99	0.04598188	0.66942149
luteolin（28）	79	0.02112834	0.60902256
Caffeic acid（37）	37	0.00552775	0.55102041
Isorhamnetin（5）	36	0.0038543	0.51428571
Quercetin-3-glucoside	31	0.00493632	0.54180602
Esculetin（16）	28	0.00397064	0.5276873
hyperin(12)	28	0.00338559	0.5276873
epicatechin	27	0.00335388	0.52090032
Protocatechuic acid（1）	21	0.00163542	0.49541284
Neochlorogenic acid（29）	19	0.00167328	0.49846154
Chlorogenic acid（22）	19	0.00167328	0.49846154
Umbelliferone（23）	18	0.00174816	0.50154799
Luteolin-7-O-β-glycoside（3）	18	0.00130885	0.49846154
stearic acid（34）	15	0.00138738	0.49390244

Supplementary Table S4: The detailed information of 15 key co-targets of STCT

Co-target	Degree	Betweenness	Closeness
HSP90AA1	68	0.01075111	0.6328125
PPARG	49	0.00499561	0.58909091
HSP90AB1	43	0.00303747	0.57651246
STAT1	41	0.00207559	0.5625
MAPT	33	0.00355197	0.54915254
PPARA	31	0.00412296	0.54915254
ALOX15	22	6.39E-04	0.53114754
RXRA	15	5.51E-04	0.51592357
